# Essential role of the D domain of linc000889 in inhibiting avian reovirus replication

**DOI:** 10.1016/j.psj.2026.107235

**Published:** 2026-06-08

**Authors:** Shaqiu Zhang, Jinkang Li, Jinghua Yang, Mingshu Wang, Renyong Jia, Shun Chen, Mafeng Liu, Dekang Zhu, Xinxin Zhao, Ying Wu, Qiao Yang, Juan Huang, Xumin Ou, Di Sun, Bin Tian, Zhen Wu, Anchun Cheng

**Affiliations:** a Avian Disease Research Center, College of Veterinary Medicine, Sichuan Agricultural University, Chengdu 611130, PR China; b Institute of Veterinary Medicine and Immunology, Sichuan Agricultural University, Chengdu 611130, PR China; c Agricultural Animal Diseases and Veterinary Public Health Key Laboratory of Sichuan Province, Sichuan Agricultural University, Chengdu 611130, PR China; d Engineering Research Center of Southwest Animal Disease Prevention and Control Technology, Ministry of Education of the PR China, Chengdu 611130, PR China; e Institute of Veterinary Immunology and Green Drugs, Veterinary Department in College of Animal Science, State Key Laboratory of Green Pesticide, Guizhou University, Guiyang 550025, PR China

**Keywords:** Avian reovirus, Linc000889, NLRX1, Avian reovirus replication

## Abstract

Avian reovirus (**ARV**) is a double-stranded RNA virus that can cause immunosuppression, irregular bleeding, spleen necrosis, and other symptoms in ducks, posing a serious threat to the poultry industry. Previous studies have confirmed that the duck-derived linc000889 can inhibit ARV replication. In this study, qRT-PCR revealed that linc000889 could promote the expression of interferon-β (**IFN-β**) and its downstream genes Mx and OASL. Subsequently, using RNA pull-down and protein mass spectrometry, we identified that the host protein NLRX1 associates with the sense strand of linc000889. This binding was verified by RNA immunoprecipitation (**RIP**) experiments. NLRX1 overexpression and knockdown experiments indicated that NLRX1 negatively regulates IFN-β expression under the conditions tested . Additionally, we found that linc000889 correlates with reduced NLRX1 protein expression. Moreover, NLRX1 complementation experiments suggested that linc000889 promotes IFN-β expression by negatively regulating NLRX1 protein, thereby inhibiting ARV replication. In this study, we also constructed eukaryotic expression plasmids lacking different domains of linc000889. The results showed that the inhibitory effect of the variant lacking the D domain on NLRX1 protein was significantly weakened, suggesting that the D domain is important for the ability for linc000889 to bind to NLRX1. In conclusion, this study confirms that the D domain is important for the ability for linc000889 to regulate NLRX1 protein, promote IFN-β expression, and inhibit ARV replication. This study provides new insights into the role of long noncoding RNAs (lncRNAs) in antiviral immune mechanisms and lays a theoretical foundation for developing lncRNAs-based antiviral strategies.

## Background

In recent years, significant progress has been made in understanding the mechanisms by which long non-coding RNAs (**lncRNAs**) regulate host immune responses. These lncRNAs can interact with DNA, RNA, and proteins to influence viral replication (Ferrè, et al., 2016; Fernandes, et al., 2019; Wang, et al., 2021; Chen, et al., 2024). LncRNAs can affect host proteins or viral proteins by directly influencing the expression of bound proteins, interfering with the molecular interactions of bound proteins themselves, or altering the cellular localization of bound proteins. For example, lncRNA HOTAIR can promote H3K27 trimethylation in the promoter regions of target genes, thereby silencing the expression of antiviral-related genes (Chen, et al., 2010; Beckedorff, et al., 2013). LncPRESS1 protects the pluripotency gene network in embryonic stem cells by binding to the SIRT6 protein and hindering its chromatin localization (Jain, et al., 2016). LncRNA LINK-A induces the tyrosine and serine phosphorylation of HIF-1α by recruiting the kinases LRRK2 and BRK, thereby stabilizing hypoxia-inducible factors and promoting a microenvironment that supports viral replication and metabolic reprogramming (Chen, et al., 2017; Peng, et al., 2021). In our early research, we identified a duck-derived lncRNA linc000889 (Zhang, et al., 2025). The present study aims to explore the mechanism by which linc000889 inhibits ARV replication and demonstrates that linc000889 associates with the host protein NLRX1. Previous studies have shown that NLRX1 can inhibit immune signaling pathways mediated by RIG-I-like receptors and Toll-like receptors, modulate the production of type I interferon (**IFN-Ⅰ**) and inflammatory factors, and play a significant role in balancing the host immune response and immunopathological damage (Soares, et al., 2013; Allen, 2014; Pickering, et al., 2021; Bi, et al., 2024). However, the role of NLRX1 in antiviral signaling remains controversial. Some studies propose that it acts as a negative regulator of innate immunity (Guo, et al., 2016a; Jiao, et al., 2021), while others suggest that it plays a positive regulatory role in the antiviral response (Lei, et al., 2016; Leber, et al., 2017). Recent studies have indicated that NLRX1 can negatively regulate interferon-β (**IFN-β**) through STING (Fritsch, et al., 2022; Xu, et al., 2023). Moreover, evidence exists that HPV16 can drive cancer immune escape via NLRX1-mediated STING degradation(Luo, et al., 2020). In zebrafish, it has been found that NLRX1 can interact with mitochondrial antiviral signaling protein (**MAVS**) through its NACHT domain, significantly impairing the host’s ability to clear grass carp reovirus and carp spring viremia virus (Zhao, et al., 2024). However, research on the function and mechanism of duck NLRX1 is still in its infancy, and its functional specificity and regulatory network in ducks require further exploration.

This study found that linc000889 can specifically bind to NLRX1 and explored the regulatory relationship between NLRX1 and IFN-β, aiming to further reveal the specific mechanism by which linc000889 inhibits the replication of ARV. Additionally, the study constructed deletion expression vectors for different secondary domains of linc000889 to identify the domains that play a major role in linc000889′s inhibition of ARV replication.

## Materials and methods

### Cell culture

In this study, all experiments were conducted using duck embryo fibroblasts (**DEF**), which were prepared from 10-day-old duck embryos purchased from a duck farm in Ya’an, Sichuan province. The tissues were digested with trypsin and the resultant cells were subsequently inoculated into a high-glucose medium enriched with 10% fetal bovine serum (**FBS**, Gibco, USA) and supplemented with 2% penicillin-streptomycin solution (Beyotime, China). Cultivation was performed under optimal conditions of 37°C and 5% CO_2_. The viral titer of the ARV strain (BioSample: SAMN00173207) was 10^-5.5^ 50% tissue culture infective dose (**TCID_50_**)/100 µL. After a 1-hour adsorption period, the cells were washed three times with PBS, then DMEM medium was added containing 2% FBS to continue culturing.

### qRT-PCR analysis

The RNAiso Plus reagent (TaKaRa, Japan) was used to efficiently extract total RNA from cells. The RNA was then reverse-transcribed into cDNA using the Hifair III 1st Strand cDNA Synthesis SuperMix for qPCR kit (Yeason, China). Quantitative analysis of target gene RNA expression was performed using the Bio-Rad CFX96 Real Time Detection System (Bio-Rad, USA), following the protocol of the ChamQ SYBR mix kit (Yeason, China). The sequences of the gene-specific primers used in the experiment are detailed in Table 1. GAPDH was selected as the internal reference gene to ensure the accuracy and reliability of the experimental results. The relative expression levels of each target RNA were calculated using the 2^−ΔΔCt^ method. Each experiment was performed in three independent biological replicates.

**Table 1:** primers sequence.

Primer	Sequence(5′-3′)
IFN-β-F	TCTACAGAGCCTTGCCTGCAT
IFN-β-R	TGTCGGTGTCCAAAAGGATGT
Mx-F	CCTAAGGGAGAAAGGACACT
Mx-R	GACCACGACACTTCACAACC
OASL-F	GCAGGCAGAGGCTGTCGTTC
OASL-R	ATGGACTCGCCGTTGGAGGA
GAPDH-F	GCAGATGCTGGTGCTGAATA
GAPDH-R	TCATGGTTCACACCCATCAC
linc000889-sense-F	**TAATACGACTCACTATAGGG**AATTCTGGAATTTCCACTTG
linc000889-sense-R	TGTTTTATCCAAATTCTTTATTCTCCAGAA
linc000889-antisense-F	AATTCTGGAATTTCCACTTG
linc000889-antisense-R	**TAATACGACTCACTATAGGG**TGTTTTATCCAAATTCTTTATTCTCCAGAA
pCA-NLRX1-F	**CATCATTTTGGCAAAGAATTC**ATGTCCCGGGCCGTGCAGGGCCGG
pCA-NLRX1-R	**TTGGCAGAGGGAAAAAGATCT**TCACAGGGTCCCGTTCTGGAGCTTCGCCAG
shRNA-NLRX1-1	GGAAGAGCACTCTCATCAAGA
shRNA-NLRX1-2	GCTTCCTGAGGCTCAACTTCA
shRNA-NLRX1-3	GCAGAAGCTCTACTTCCAGAT
shRNA-NLRX1-4	GCTGTTCAAAGAGGAGGACTA
pCA-linc000889-MutA1/B1/C1/D1/E1-F (equivalent to pCA-linc000889-F)	**CATCATTTTGGCAAAGAATTC**AATTCTGGAATTTCCACTTGGGCT
pCA-linc000889-MutA1-R	CACAGTCCCTGGGCCTGCCATCC
pCA-linc000889-MutA2-F	GATGGCAGGCCCAGGGACTGTGGGA
pCA-linc000889-MutA2/B2/C2/D2/E2-R (equivalent to pCA-linc000889-R)	**TTGGCAGAGGGAAAAAGATCT**TGTTTTATCCAAATTCTTTATTCTCCAGAATCATAATT
pCA-linc000889-MutB1-R	GCAGCTACTCATGACAGCAGCTGTGCT
pCA-linc000889-MutB2-F	GTCATGAGTAGCTGCACGATACCA
pCA-linc000889-MutC1-R	TCAGGATGACAAGACTCGTTCTGCAGGTATCTTTGCACCCA
pCA-linc000889-MutC2-F	TGCAAAGATACCTGCAGAACGAGTCTTGTCATCCTGAGCCCCTG
pCA-linc000889-MutD1-R	GCGCAGTCCGTGCCCTTCCCACAGT
pCA-linc000889-MutD2-F	GAAGGGCACGGACTGCGCCTCTATTGTT
pCA-linc000889-MutE1-R	CATCTATCTCATATGAAAAGAACAATAGAGGCGCAGTCCCA
pCA-linc000889-MutE2-F	TGCGCCTCTATTGTTCTTTTCATATGAGATAGATGGTATTTTCATCGGTGGATCAT
pCA-linc000889-MutF-F	**CATCATTTTGGCAAAGAATTC**AATTCTGGAATTTCCACTTGGGCTTCTCTG
pCA-linc000889-MutF-R	**TTGGCAGAGGGAAAAAGATCT**TGTTTTATCCAAATTCTTTATTCTCAGCTGCCCTCC
linc000889-MutD-F	CACGGACTGCGCCTCTATT
linc000889-MutD-R	GGAGACTCTGAGCTGCCC

Note: The T7 promoter sequence, the homologous arm sequence of the primers, and the corresponding enzyme digestion site sequence (underlined) are indicated in bold font.

### Plasmid construction and transfection

The successfully amplified full-length fragment of NLRX1 was then inserted into the pCAGGS vector (Addgene, USA) using homologous recombination (Vazyme, China). Thus, we successfully obtained the experimental plasmid pCA-NLRX1 and the control plasmid pCAGGS. We also commissioned GenePharma (China) to synthesize shRNA targeting NLRX1. From this, we obtained four shRNAs targeting NLRX1 expression: shRNA-NLRX1-1, shRNA-NLRX1-2, shRNA-NLRX1-3 and shRNA-NLRX1-4, as well as the control shRNA-NC. DEFs were transfected using Lipofectamine 2000 (Yeason, China) after being plated in a 12-well plate for 12 to 24 hours, when cell confluence reached approximately 80%-90%.

### Virus titer determination

Samples were collected at various time points, and the cells underwent two freeze-thaw cycles. The virus sample was then centrifuged and stored at -80°C. Viral suspensions were serially diluted 10‑fold from 10^-1^ to 10^-8^. For the range between 10^-3^ and 10^-4^, an additional 5-fold dilution was included. Thus the full dilution series consisted of 10^-1^, 10^-2^, 10^-3^, 10^-3.5^, 10^-4^, 10^-4.5^, 10^-5^, 10^-6^, 10^-7^, and 10^-8^. Each dilution was dispensed into eight replicate wells of a 96-well cell culture plate, with 100 µL of diluted viral suspension added per well. Following this, 100 µL of a passaged cell suspension was added to each well, and the plates were incubated at 37°C with 5% CO₂ for 5-7 days. Cytopathic effects (**CPE**), characterized by loss of the original fibrous morphology, progressive irregularity of cellular structures and shapes, indistinct cell boundaries, and eventual cell detachment, were observed and documented. The viral titer, expressed as TCID₅₀, was calculated using the Reed-Muench method. Each experiment was performed in three independent biological replicates.

### Western blot

We lysed cell cultures at 4°C using a RIPA lysis buffer (Beyotime, China), supplemented with 1 mM PMSF (Beyotime, China), to extract proteins. The denatured protein samples were resolved by SDS-PAGE and transferred onto 0.45 µm PVDF membranes (Millipore, USA). After blocking the membranes in 5% skim milk in Tris-buffered saline with tween-20 (**TBST**) for 2 hours, we washed them with TBST containing 0.1% Tween 20. Following the manufacturer's protocol, we incubated the membranes with primary antibodies diluted appropriately overnight at 4°C: anti-ARV-σc antibody (Prepared in this study, 1:1000), anti-GAPDH antibody (Proteintech, China, 1:5000), and anti-NLRX1 antibody (Abclonal, China, 1:2000). After incubation, the membranes were washed three times with TBST and then incubated with goat anti-mouse or anti-rabbit secondary antibodies (Abclonal, China, 1:10000) at 37°C for 60 min. Subsequent washing was followed by treatment with ECL reagents (Thermo Fisher Scientific, USA). Proteins bound to the membrane were visualized and analyzed using the Touch Imager (e-BLOT, China). GAPDH served as the internal reference protein, with each experiment conducted in triplicate. ImageJ software was utilized for grayscale analysis to assess the differential expression of the proteins of interest.

### RNA pull-down

Approximately 6 × 10^7^ cells were collected, and lysis buffer was added before sonication (Gene Create, China). After centrifugation, the supernatant was collected and divided into the Sense group, Antisense group and the Input group. Streptomycin affinity magnetic beads were washed three times with Wash Buffer I to remove impurities. Biotin-labeled RNA probes (200 pmol) and 1 × RNA binding buffer were added to the magnetic bead tube and incubated for 2 hours at room temperature to form the RNA-magnetic bead complex. The cell lysate supernatants were then incubated with the RNA-bead complexes overnight at 4°C. After incubation, the samples were washed three times sequentially with Wash Buffer I and Wash Buffer II. Unbound samples were removed using magnetic rack separation. The magnetic beads and eluates were collected separately and stored at -20°C for subsequent experiments.

### Protein mass spectrometry detection and protein silver staining

The magnetic beads obtained after RNA pull-down were eluted with PBS and sent to Hangzhou Jingjie Biotechnology Co., Ltd. for mass spectrometry detection. The eluent from the RNA pull-down was mixed with 5 × SDS loading buffer, boiled in water for 10 minutes, and then subjected to SDS-PAGE electrophoresis to separate the elution products. The binding proteins were verified by silver staining (Gene Create, China).

### RNA immunoprecipitation (RIP)

About 6 × 10^7^ cells were collected and divided into three groups: IP group, IgG group and Input group after adding RIP buffer and sonication (Gene Create, China). Protein A/G magnetic beads were divided into two portions, one for the IgG group and the other for the IP group. The magnetic beads were washed three times with RIP wash buffer. Next, 3 µg of the target antibody was added to the IP tube, while an equal amount of homologous IgG was added to the IgG tube. The samples were incubated with rotation for 2 hours at room temperature to allow the antibodies to bind to the magnetic beads. The beads were then washed three times to remove nonspecifically bound antibodies. RIP buffer and cell lysate were added to the IgG and IP tubes, respectively, and the samples were incubated overnight at 4°C to form RNA-protein complexes. Subsequently, the magnetic beads were separated using a magnetic frame and washed five times with RIP wash buffer. Finally, the magnetic beads were resuspended for subsequent RIP-qPCR analysis.

### Dual luciferase assay

A luciferase reporter plasmid (IFN-β-Luc, 400 ng), a Renilla luciferase expression plasmid (pRL-TK, 40 ng), and gradient doses of plasmid pCAGGS or experimental group plasmids (including pCA-NLRX1, RIG-Flag, MDA5-Flag, STING-Myc, TBK1-HA, and IRF7-Myc) were transfected into DEF cells that were in good growth conditions. Three biological replicates were set for each group. Firefly luciferase and Renilla luciferase activities were detected using a Dual-Luciferase Reporter Assay System (Thermo Fisher, USA), and statistical results were obtained according to the formula: fold change in normalized reporter activity= (ratio of the experimental group)/ (ratio of the control group) (Yeason, China).Ratio calculation: The firefly/Renilla ratio was calculated for each well to normalize for well-to-well variations in transfection efficiency and cell viability.The control group is very close to 1, while the experimental group is represented by a multiple change relative to the control group.

## Results

### Linc000889 promotes IFN-β expression

**Fig. 1:**
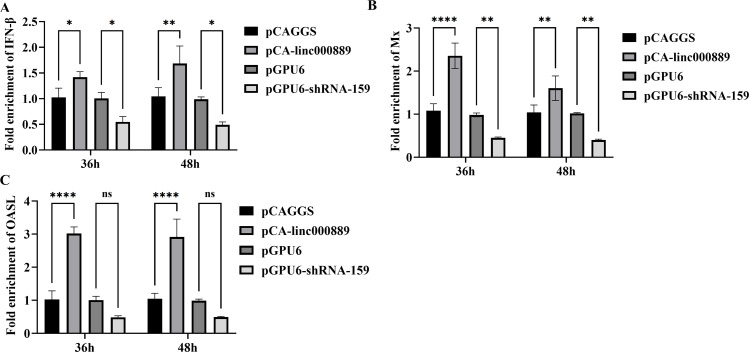
**Effect of linc000889 on IFN-β, Mx and OASL expression** (A-C) 12-well plates were transfected with 1 µg (pGPU6 and pGPU6-shRNA-159) or 2 µg (pCAGGS and pCA-linc000889) plasmid for 24 h DEF cells were infected with ARV at an MOI of 0.5, and samples were collected at 36 h and 48 h post-infection. Transcription levels of *IFN-β, Mx* and *OASL* were detected. All experiments were performed in three independent biological replicates. Data are shown as mean ± SEM (n=3). Statistical significance: ns, P > 0.05; *, P <0.05; **, P <0.01; ***, P <0.001; ****, P<0.0001.

After overexpression of linc000889, qRT-PCR was used to analyze the transcriptional changes of IFN-β, Mx, and OASL. The results indicated that linc000889 significantly promoted the expression of IFN-β, Mx, and OASL. And after knocking down the expression of linc000889, the expression of IFN-β, Mx, and OASL was significantly reduced. These findings demonstrate that linc000889 can enhance the transcriptional expression of IFN-β and its downstream genes Mx and OASL (Fig. 1).

### Linc000889 binds and suppresses NLRX1 protein

**Fig. 2:**
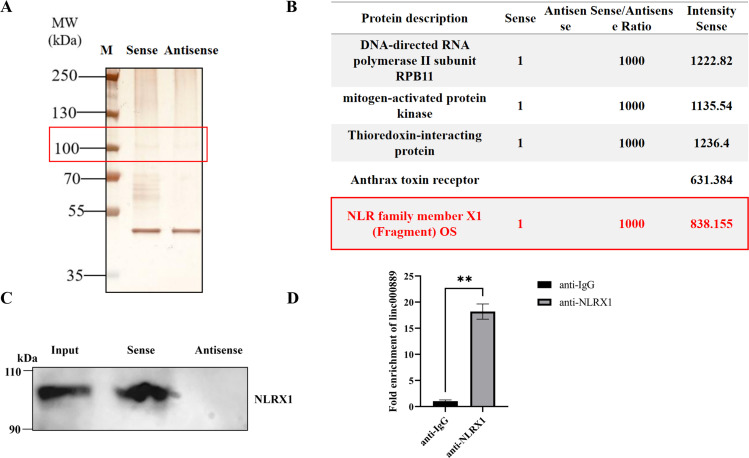
L**inc000889 associates with the NLRX1 protein** (A) Silver staining of RNA pull-down eluates. The red box indicates a band at 108 kDa present in the sense group but absent in the antisense group. (B) Mass spectrometry identification of proteins bound to linc000889 (partial list shown). (C) Western blot validation of the association between linc000889 and NLRX1. Sense: sense RNA probe; Antisense: antisense RNA probe. (D) RIP-qPCR analysis of NLRX1 association with linc000889. All experiments were performed in three independent biological replicates. Data are shown as mean ± SEM (n=3). Statistical significance: **P < 0.01.

The study utilized RNA pulldown and protein mass spectrometry techniques to explore host proteins that specifically bind to linc000889. The red box in Figure A indicates the presence of a protein band around 108 kDa in the sense RNA probe group, but not in the antisense RNA probe group (Fig. 2A; Sup. 1). The protein mass spectrometry detection results showed NLRX1 binding in the sense RNA probe group, but not in the antisense RNA probe group (Fig. 2B). Western blot analysis was performed on the RNA pull-down eluent, and the results showed that the Input group and the sense RNA probe group could bind to NLRX1 protein, while the antisense RNA probe group could not (Fig. 2C). The sense RNA probe of linc000889 specifically enriched NLRX1. After the RIP experiment, qRT-PCR was used to detect the transcriptional expression levels of linc000889 in the experimental group (anti-NLRX1) and the control group (anti IgG). The results showed that the binding ability of linc000889 in the experimental group was significantly enhanced (Fig. 2D).

### Linc000889 inhibits NLRX1 expression

**Fig. 3:**
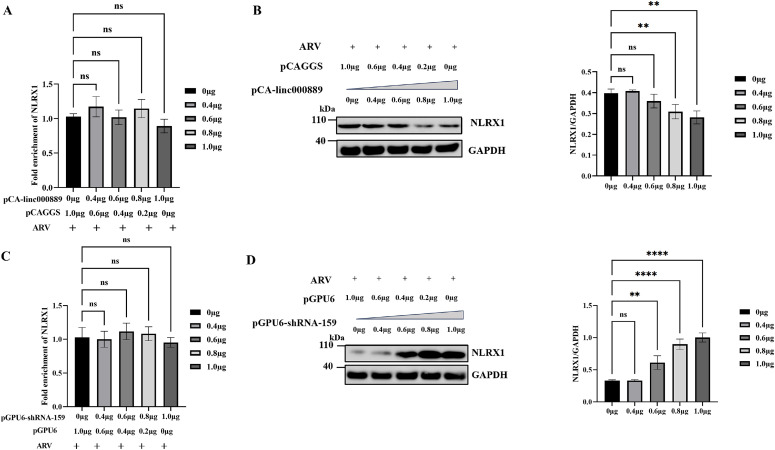
L**inc000889 inhibited NLRX1 protein expression** (A) DEF cells in 24-well plates were transfected with increasing doses of pCA-linc000889, then infected with ARV at an MOI of 0.5 for 24 h. Samples were collected 36 h later for qRT-PCR analysis of NLRX1 mRNA. (B) Western blot analysis of NLRX1 protein under the same conditions. (C) DEF cells were transfected with increasing doses of pGPU6-shRNA-159, then infected with ARV at an MOI of 0.5 for 24 h. Samples were collected 36 h later for qRT-PCR analysis of NLRX1 mRNA. (D) Western blot analysis of NLRX1 protein under the same conditions. All experiments were performed in three independent biological replicates. Data are shown as mean ± SEM (n=3). Statistical significance: ns, P > 0.05; *, P < 0.05; **, P < 0.01.

Transfecting pCA-linc000889 at different doses, the effect of linc000889 on the transcription and translation levels of NLRX1 was detected. The experimental results showed that linc000889 had no significant effect on the transcription level of NLRX1, but could inhibit the expression level of NLRX1 protein (Fig. 3A & B). The dose-dependent knockdown experiment of linc000889 indicated that knocking down linc000889 can promote the expression of NLRX1 protein (Fig. 3C & D). Linc000889 can inhibit the expression of NLRX1 protein.

### Negative regulation of IFN-β expression by NLRX1

**Fig. 4:**
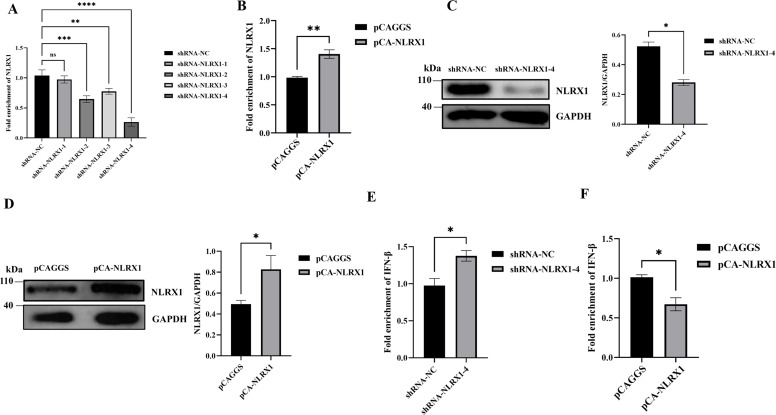
**NLRX1 negatively regulates IFN-β expression under the conditions tested in DEF cells** (A) Knockdown efficiency of four shRNAs targeting NLRX1, assessed by qRT-PCR. (B) Overexpression efficiency of pCA-NLRX1, assessed by qRT-PCR. (C) Western blot validation of NLRX1 knockdown by shRNA-NLRX1-4. (D) Western blot validation of NLRX1 overexpression by pCA-NLRX1. (E) qRT-PCR analysis of endogenous IFN-β mRNA after NLRX1 knockdown. (F) qRT-PCR analysis of endogenous IFN-β mRNA after NLRX1 overexpression. For the dose series, empty pCAGGS was added to bring total DNA to 1.0 µg per well. The shRNA dose series was similarly balanced with empty pGPU6. All experiments were performed in three independent biological replicates. Data are shown as mean ± SEM (n=3). Statistical significance: ns, P > 0.05; *, P < 0.05; **, P < 0.01; ***, P < 0.001; ****, P < 0.0001.

**Fig. 5:**
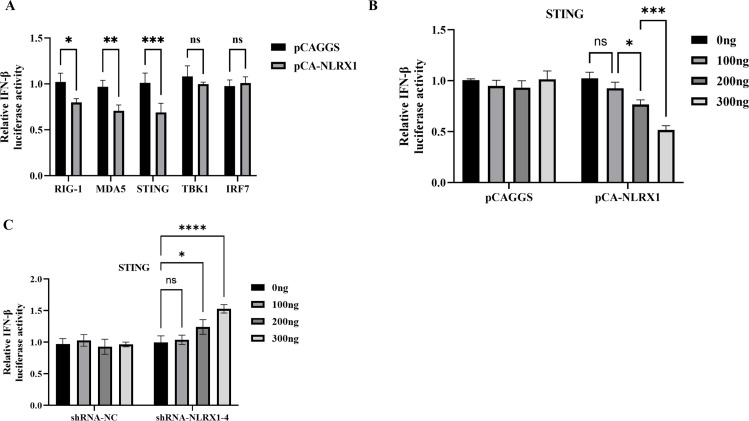
**NLRX1 inhibits RIG‑I, MDA5, and STING‑mediated but not TBK1 or IRF7‑mediated IFN‑β promoter activation** (A) DEF cells in 24-well plates were co-transfected with IFN-β-Luc (400 ng), pRL-TK (40 ng), pCAGGS or pCA-NLRX1 (200 ng), and the indicated signaling molecule plasmids (200 ng). After 24 h, cells were infected with ARV (MOI=0.5) for 36 h, and IFN-β promoter activity was measured by dual-luciferase assay. (B) Dose-dependent inhibition of STING-induced IFN-β-Luc activity by increasing amounts of pCA-NLRX1. (C) Dose-dependent relief of STING-induced IFN-β-Luc activity by increasing amounts of shRNA-NLRX1-4. All experiments were performed in three independent biological replicates. Data are shown as mean ± SEM (n=3). Statistical significance: ns, P > 0.05; *, P < 0.05; **, P < 0.01; ***, P < 0.001; ****, P < 0.0001.

The study first validated the knockdown and overexpression efficiency of shRNA-NLRX1 and pCA-NLRX1 on DEF through qRT-PCR and western blot experiments. The results showed that pCA-NLRX1 was successfully expressed in DEF cells, and shRNA-NLRX1-4 had a higher knockdown efficiency. Therefore, it was selected for subsequent experiments for further functional exploration of NLRX1 in the study (Fig. 4A - D). qRT-PCR experiments showed that knocking down NLRX1 significantly increased the expression of IFN-β, which was also confirmed by overexpression experiments (Fig. 4E & F). The study investigated whether NLRX1 affects duck RLR-IFN signaling using a dual-luciferase reporter assay. The results showed that overexpression of NLRX1 significantly reduced the activation of IFN-β-Luc by RIG-I, MDA5, and STING, but did not affect the activation of IFN-β-Luc by TBK1 and IRF7 (Fig. 5A), indicating that NLRX1 may act upstream of TBK1 and IRF7. Co transfection of pCA-NLRX1 or shRNA-NLRX1-4 and STING-Myc plasmids at different doses showed a decrease in IFN-β promoter activity with increasing NLRX1 (Fig. 5B & C). This suggests that NLRX1 may negatively regulate the activity of the IFN - β promoter through STING, thereby inhibiting the expression of IFN-β. The above results indicate a negative regulatory relationship between NLRX1 and IFN-β.

### Linc000889 negatively regulates NLRX1 protein to promote IFN-β expression and inhibit ARV replication

**Fig. 6:**
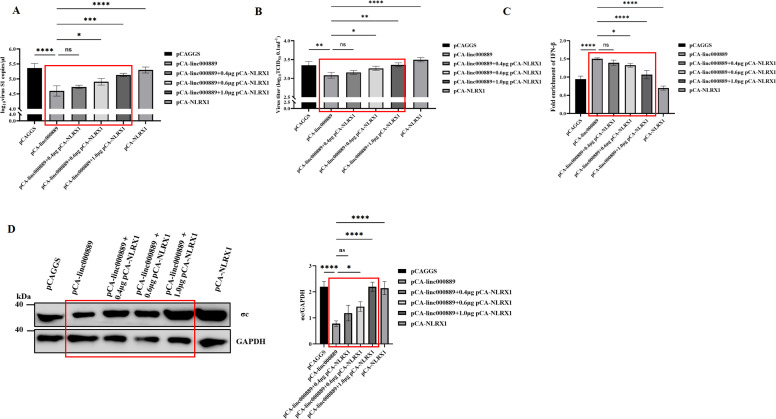
L**inc000889 negatively regulates NLRX1 protein to promote IFN-β expression and inhibit ARV replication** (A) DEF cells in 12-well plates were co-transfected with pCA-linc000889 and increasing doses of pCA-NLRX1, then infected with ARV (MOI=0.5) for 24 h. Samples were collected 36 h later for qRT-PCR analysis of S1 gene copy number. (B) Viral titers (TCID₅₀) determined under the same conditions. (C) Western blot analysis of σc protein expression. (D) qRT-PCR analysis of IFN-β expression. All experiments were performed in three independent biological replicates. Data are shown as mean ± SEM (n=3). Statistical significance: ns, P > 0.05; *, P < 0.05; **, P < 0.01; ***, P < 0.001; ****, P < 0.0001.

To investigate whether linc000889 promotes IFN-β expression through NLRX1 protein, an NLRX1 complementation experiment was designed. The co transfection of pCA-lin000889 and different doses of pCA-NLRX1 was used as the experimental group, the pCAGGS transfection group was used as the blank control group, and the pCA-linc000889 and pCA-NLRX1 transfection groups were used as the negative control group and positive control group, respectively. The experimental results showed that with the increase of NLRX1 complementation degree, S1 copy number increased progressively (Fig. 6A), the virus titer continued to increase (Fig. 6B), and the expression level of σc protein continued to increase (Fig. 6C). Meanwhile, the qRT-PCR experiment results showed that as the amount of NLRX1 re-expression increased, the expression level of IFN-β continued to decrease (Fig. 6D). The above experimental results demonstrate that NLRX1 supplementation can partially counteract the inhibitory effect of linc000889 on ARV replication and increase ARV replication. Linc000889 promotes IFN‑β expression, at least in part, through reducing NLRX1 protein.

### The D domain is an important region for linc000889 to bind with NLRX1

**Fig. 7:**
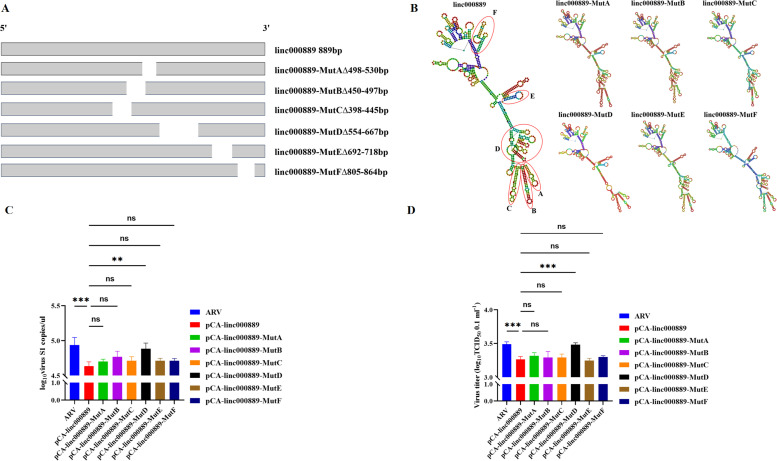
**Deletion of the D domain of linc000889 impairs its antiviral activity** (A) Schematic diagram of linc000889 deletion mutants (MutA‑MutF). (B) Predicted secondary structures of the deletion mutants. (C) DEF cells in 24-well plates were transfected with 1 µg of mutant plasmid for 24 h, then infected with ARV at an MOI of 0.5. Samples were collected 36 h post-infection for qRT-PCR analysis of S1 gene copy number. (D) ARV titers (TCID₅₀) under the same conditions. All experiments were performed in three independent biological replicates. Data are shown as mean ± SEM (n=3). Statistical significance: ns, P > 0.05; *, P < 0.05; **, P < 0.01; ***, P < 0.001; ****, P < 0.0001.

**Fig. 8:**
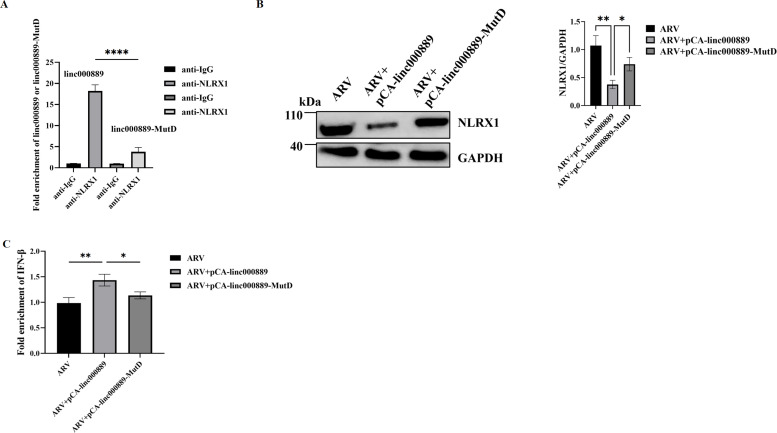
**The D domain of linc000889 is critical for NLRX1 binding and subsequent IFN‑β induction** (A) RIP-qPCR analysis comparing the binding ability of wild-type linc000889 and linc000889-MutD to NLRX1. (B) Western blot analysis of NLRX1 protein expression in cells transfected with wild-type or MutD linc000889 and then infected with ARV. (C) qRT-PCR analysis of IFN-β mRNA expression under the same conditions. All experiments were performed in three independent biological replicates. Data are shown as mean ± SEM (n=3). Statistical significance: ns, P > 0.05; *, P < 0.05; **, P < 0.01; ***, P < 0.001; ****, P < 0.0001.

Six stem-loop deletion mutants were generated of linc000889 and construction of corresponding variant expression plasmids pCA-linc000889-MutA, pCA-linc000889-MutB, pCA-linc000889-MutC, pCA-linc000889-MutD, pCA-linc000889-MutE and pCA-linc000889-MutF (Fig. 7A & B). After transfecting the corresponding plasmid into DEF, virus copy number and virus titer were detected, and it was found that the inhibitory effect of the missing stem loop domain D on virus replication was significantly weakened (Fig. 7C & D). RIP qPCR experiments have shown that the anti-NLRX1 group has a significantly stronger ability to bind linc000889 than linc000889-MutD (Fig. 8A); Western blot experiments showed that compared with the group transfected with pCA-linc000889, the group transfected with pCA-linc000889-MutD significantly reduced its inhibitory effect on NLRX1 protein (Fig. 8B); qRT-PCR experiments showed that the promotion effect of IFN - β was significantly reduced in the pCA-linc000889-MutD transfection group compared to the pCA-linc000889 transfection group (Fig. 8C). These data suggest that the D domain is an important region for the binding of linc000889 and NLRX1.

## Discussion

This study has identified that linc000889 can promote the expression of host antiviral signaling molecules, including IFN-β, OASL, and Mx. To elucidate the underlying mechanisms by which linc000889 affects the expression of these antiviral signaling molecules and inhibits ARV replication, further research was conducted. Previous studies have indicated that lncRNAs can modulate host antiviral responses by directly binding to or indirectly regulating key molecules in pathways such as RIG-I, MAVS, STING, TBK1, and IRF3/IRF7 (Wang, et al., 2018; Pan, et al., 2019; John, et al., 2024). For instance, lncRNA ISR enhances viral RNA recognition by binding to RIG-I, thereby significantly promoting type I IFN production (Fernandes, et al., 2019). LncLrrc55 AS drives IRF3 signaling via a forward feedback loop, promoting sustained IFN-I expression (Zhou, et al., 2019). LncRNA GM enhances TBK1 kinase activity and TBK1-mediated phosphorylation of IRF3, thereby affecting IFN-I expression (Wang, et al., 2020). LncRNA MALAT1 reduces antiviral ability and inhibits autoimmune diseases by suppressing the production of type I interferons (Liu, et al., 2020). Additionally, lncRNAs can regulate downstream immune effects on viral replication by binding to host proteins. For example, recent studies have shown that LINC0143 interacts with PRMT1 (protein arginine methyltransferase) to inhibit PRMT1 ubiquitination and degradation, thereby promoting the binding of PRMT1 to HBV cccDNA and inhibiting HBV replication (Fang, et al., 2022). Additionally, lncITPRIP-1 can inhibit HCV replication by promoting MDA5 activation (Xie, et al., 2018).

In this study, the host protein NLRX1, which associates with linc000889, was identified through RNA pulldown, protein mass spectrometry, and RIP experiments. NLRX1, a member of the NOD-like receptor family, has been reported to negatively regulate interferon production in mammals (Xia, et al., 2011), but its function in ducks has been less well studied. ARV infection is likely to trigger various cellular stresses (endoplasmic reticulum stress, oxidative stress, inflammatory stress), which may alter the rates of protein synthesis, protein folding, or protein degradation. This study demonstrated for the first time the negative regulatory effect of NLRX1 on IFN-β in DEF cells and showed using a dual-luciferase reporter assay that it may inhibit IFN-β promoter activity via STING. Further work is needed regarding how duck NLRX1 protein affects STING and inhibits IFN-β promoter activity. Meanwhile, this study revealed that linc000889 can inhibit NLRX1 protein expression. Dose-based replenishment experiments of NLRX1 have indicated that linc000889 promotes IFN-β expression and inhibits ARV replication by negatively regulating NLRX1 protein. The mechanism by which linc000889 reduces NLRX1 protein abundance remains unclear in this study. However, three hypotheses have been proposed: firstly, linc000889 may recruit E3 ubiquitin ligase to catalyze the ubiquitination modification of NLRX1, allowing it to be recognized and degraded by the proteasome; secondly, linc000889 may activate selective autophagy to promote the encapsulation of NLRX1 into autophagosomes and its degradation after fusion with lysosomes; thirdly, after linc000889 binds to NLRX1, its interaction with molecular partners (such as HSP90) may be disrupted, leading to structural instability of NLRX1 and its clearance by cytoplasmic quality control systems (such as the ubiquitination system). We plan to report the specific mechanism in our upcoming research. Nonetheless, this study has indicated that linc000889 can negatively regulate NLRX1 protein to promote IFN-β expression and inhibit ARV replication (this does not prove that NLRX1 is the sole mediator; other NLRX1-independent pathways may also contribute).

Next, further research will focus on exploring the structural domains that play a major role in linc000889′s antiviral function. The functions of many lncRNAs depend on their secondary structures, which is composed of multiple functional domains that form stable spatial conformations through intramolecular base pairing (Guo, et al., 2016b). Among these, the stem-loop structure, as a key element of the secondary structure of lncRNAs, plays a significant role in post-transcriptional regulation of gene expression, protein-RNA interactions, cell signal transduction, and other biological processes (Chen, 2022). For example, the stem-loop structure of SRA can interact with the steroid receptor activator complex, enhancing its transcriptional activation function (Novikova, et al., 2012). The stem-loop structure of SPRY4-IT1 regulates its localization and stability within cells by binding to specific transcription factors, thereby affecting the proliferation and migration of cancer cells (Khaitan, et al., 2011). The stem-loop structure of HOTAIR can guide the PRC2 complex to target specific gene promoter regions, leading to gene silencing (Tsai, et al., 2010). In this study, RNAfold prediction results indicated that the D domain contains three highly conserved stem-loop structures, which may provide specific binding sites for protein interactions. However, we did not conduct direct structural analysis to rule out global folding changes. Compared to variants lacking other domains, the linc000889 variant lacking the D domain significantly reduced its inhibitory effect on ARV replication. We speculate that this is because the linc000889 variant lacking the D domain has lower affinity for NLRX1, significantly reducing its inhibitory effect on NLRX1 protein and thereby promoting IFN-β expression. qPCR and western blot experiments confirmed the previous hypothesis. It is worth noting that deletion of other domains (MutB and MutE) also moderately reduced the antiviral activity, suggesting that multiple regions may be involved in the function of linc000889, with the D domain playing the major role. Similar mechanisms have also been reported in related studies. Studies have shown that the four long arm rings of lncRNA#61 mediate its broad-spectrum antiviral effect primarily by inhibiting the polymerase activity of the influenza virus and the nuclear aggregation of key polymerase components (Hu, et al., 2023). The stem-loop structure of lncRNA#45 can inhibit the polymerase activity and nuclear accumulation of NP and PA, thereby inhibiting influenza virus replication (Zhang, et al., 2021). These findings collectively suggest that the specific secondary domains of lncRNAs may play a universal and critical role in antiviral function, providing an important theoretical basis and experimental direction for further in-depth research on the functional mechanisms of lncRNAs.

In summary, deletion of the D domain weakens the ability of linc000889 to suppress NLRX1, induce IFN-β, and restrict ARV replication, thereby weakening its inhibitory effect on ARV replication.

## Data availability statement

The datasets presented in this study can be found in online repositories. The names of the repository/repositories and accession number(s) can be found in the article/Supplementary material.

## Ethics statement

The animal study was approved by Animal Welfare Committee, Sichuan Agricultural University. The study was conducted in accordance with the local legislation and institutional requirements.

## Acknowledgements

The author(s) declare that financial support was received for the research, authorship, and/or publication of this article. This work was supported by National Key Research and Development Program of China (2024YFF1000900), China Agriculture Research System of MOF and MARA (CARS-41-17), Sichuan Veterinary Medicine and Drug Innovation Group of China Agricultural Research System (SCCXTD-2026-18). We would like to thank Mafeng Liu, Renyong Jia, and Shun Chen for their assistance with cell culture, virus infection, viral titer detection, and qRT-PCR assays. We are also grateful to Ying Wu and Xumin Ou for performing plasmid transfection, Western blot, and dual-luciferase reporter assays, and to Dekang Zhu for analyzing the experimental statistical data. In addition, we sincerely thank Xinxin Zhao for validation of core experimental results. We also appreciate the general support provided by Qiao Yang during the course of this study. All the above-mentioned individuals are affiliated with Research Center of Avian Diseases, College of Veterinary Medicine, Sichuan Agricultural University, Chengdu, Sichuan, P.R. China.

## Generative AI statement

The authors declare that no Gen AI was used in the creation of this manuscript.

## Publisher’s note

All claims expressed in this article are solely those of the authors and do not necessarily represent those of their affiliated organizations, or those of the publisher, the editors and the reviewers. Any product that may be evaluated in this article, or claim that may be made by its manufacturer, is not guaranteed or endorsed by the publisher.

## CRediT authorship contribution statement

**Shaqiu Zhang:** Writing – review & editing, Writing – original draft, Visualization, Validation, Formal analysis, Data curation, Conceptualization, Funding acquisition. **Jinkang Li:** Writing – review & editing, Writing – original draft, Formal analysis, Data curation. **Jinghua Yang:** Investigation, Formal analysis, Data curation. **Mingshu Wang:** Formal analysis, Data curation, Validation. **Renyong Jia:** Resources. **Shun Chen:** Resources. **Mafeng Liu:** Resources. **Dekang Zhu:** Resources. **Xinxin Zhao:** Resources. **Ying Wu:** Resources. **Qiao Yang:** Resources. **Juan Huang:** Formal analysis, Data curation, Validation. **Xumin Ou:** Resources. **Di Sun:** Investigation, Data curation, Validation. **Bin Tian:** Data curation, Validation, Visualization. **Zhen Wu:** Data curation, Resources, Supervision, Investigation, Funding acquisition. **Anchun Cheng:** Writing – review & editing, Writing – original draft, Supervision, Resources, Project administration, Conceptualization, Funding acquisition.

## Disclosures

The authors declare that they have no known competing financial interests or personal relationships that could have appeared to influence the work reported in the present study.

## Appendix Supplementary materials

Image, application 1

Image, application 2

Image, application 3

Image, application 4

Image, application 5

Image, application 6
