# Supplementary material for: Essential role of the D domain of linc000889 in inhibiting avian reovirus replication
Source: Poult Sci. 2026 Jun 8;105(10):107235. doi: 10.1016/j.psj.2026.107235 (PMC13316747; doi:10.1016/j.psj.2026.107235)
Supplement: Supplementary file 1 [file mmc1.docx]

**Table 1 primers sequence**

| Primer | Accession No. | Sequence(5'-3') | bp |
| --- | --- | --- | --- |
| IFN-β-F | [NM_001310827.2](https://www.ncbi.nlm.nih.gov/nucleotide/NM_001310827.2?report=genbank&log$=nucltop&blast_rank=3&RID=0FKT64X7014) | TCTACAGAGCCTTGCCTGCAT |  |
| IFN-β-R |  | TGTCGGTGTCCAAAAGGATGT |  |
| Mx-F | [XM_035545890.2](https://www.ncbi.nlm.nih.gov/nucleotide/XM_035545890.2?report=genbank&log$=nucltop&blast_rank=1&RID=0FMGWJPD016) | CCTAAGGGAGAAAGGACACT |  |
| Mx-R |  | GACCACGACACTTCACAACC |  |
| OASL-F | [XM_032194146.1](https://www.ncbi.nlm.nih.gov/nucleotide/XM_032194146.1?report=genbank&log$=nucltop&blast_rank=1&RID=0FNUVC7Z014) | GCAGGCAGAGGCTGTCGTTC |  |
| OASL-R |  | ATGGACTCGCCGTTGGAGGA |  |
| GAPDH-F | [XM_056481862.1](https://www.ncbi.nlm.nih.gov/nucleotide/XM_056481862.1?report=genbank&log$=nucltop&blast_rank=1&RID=0SKC2T01014) | GCAGATGCTGGTGCTGAATA |  |
| GAPDH-R |  | TCATGGTTCACACCCATCAC |  |
| linc000889-sense-F |  | **TAATACGACTCACTATAGGG**AATTCTGGAATTTCCACTTG | 889 |
| linc000889-sense-R |  | TGTTTTATCCAAATTCTTTATTCTCCAGAA |  |
| linc000889-antisense-F |  | AATTCTGGAATTTCCACTTG | 889 |
| linc000889-antisense-R |  | **TAATACGACTCACTATAGGG**TGTTTTATCCAAATTCTTTATTCTCCAGAA |  |
| pCA-NLRX1-F |  | **CATCATTTTGGCAAAGAATTC**ATGTCCCGGGCCGTGCAGGGCCGG |  |
| pCA-NLRX1-R |  | **TTGGCAGAGGGAAAAAGATCT**TCACAGGGTCCCGTTCTGGAGCTTCGCCAG |  |
| shRNA-NLRX1-1 |  | GGAAGAGCACTCTCATCAAGA |  |
| shRNA-NLRX1-2 |  | GCTTCCTGAGGCTCAACTTCA |  |
| shRNA-NLRX1-3 |  | GCAGAAGCTCTACTTCCAGAT |  |
| shRNA-NLRX1-4 |  | GCTGTTCAAAGAGGAGGACTA |  |
| pCA-linc000889-MutA1/B1/C1/D1/E1-F（equivalent to pCA-linc000889-F） |  | **CATCATTTTGGCAAAGAATTC**AATTCTGGAATTTCCACTTGGGCT |  |
| pCA-linc000889-MutA1-R |  | CACAGTCCCTGGGCCTGCCATCC | 856 |
| pCA-linc000889-MutA2-F |  | GATGGCAGGCCCAGGGACTGTGGGA | 360 |
| pCA-linc000889-MutA2/B2/C2/D2/E2-R（equivalent to pCA-linc000889-R） |  | **TTGGCAGAGGGAAAAAGATCT**TGTTTTATCCAAATTCTTTATTCTCCAGAATCATAATT |  |
| pCA-linc000889-MutB1-R |  | GCAGCTACTCATGACAGCAGCTGTGCT | 449 |
| pCA-linc000889-MutB2-F |  | GTCATGAGTAGCTGCACGATACCA | 393 |
| pCA-linc000889-MutC1-R |  | TCAGGATGACAAGACTCGTTCTGCAGGTATCTTTGCACCCA | 397 |
| pCA-linc000889-MutC2-F |  | TGCAAAGATACCTGCAGAACGAGTCTTGTCATCCTGAGCCCCTG | 445 |
| pCA-linc000889-MutD1-R |  | GCGCAGTCCGTGCCCTTCCCACAGT | 553 |
| pCA-linc000889-MutD2-F |  | GAAGGGCACGGACTGCGCCTCTATTGTT | 223 |
| pCA-linc000889-MutE1-R |  | CATCTATCTCATATGAAAAGAACAATAGAGGCGCAGTCCCA | 691 |
| pCA-linc000889-MutE2-F |  | TGCGCCTCTATTGTTCTTTTCATATGAGATAGATGGTATTTTCATCGGTGGATCAT | 172 |
| pCA-linc000889-MutF-F |  | **CATCATTTTGGCAAAGAATTC**AATTCTGGAATTTCCACTTGGGCTTCTCTG | 831 |
| pCA-linc000889-MutF-R |  | **TTGGCAGAGGGAAAAAGATCT**TGTTTTATCCAAATTCTTTATTCTCAGCTGCCCTCC |  |
| linc000889-MutD-F |  | CACGGACTGCGCCTCTATT |  |
| linc000889-MutD-R |  | GGAGACTCTGAGCTGCCC |  |

Note: The T7 promoter sequence, the homologous arm sequence of the primers, and the corresponding enzyme digestion site sequence (underlined) are indicated in bold font.
