# Supplementary material for: Essential role of the D domain of linc000889 in inhibiting avian reovirus replication
Source: Poult Sci. 2026 Jun 8;105(10):107235. doi: 10.1016/j.psj.2026.107235 (PMC13316747; doi:10.1016/j.psj.2026.107235)
Supplement: Supplementary file 3 [file mmc3.pdf]

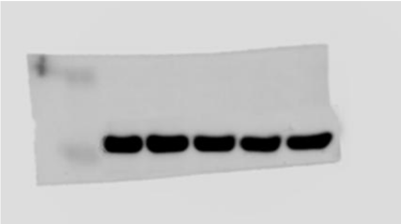	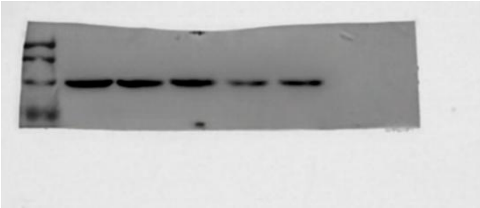
FIG3-B, anti-GAPDH	FIG3-B, anti-NLRX1
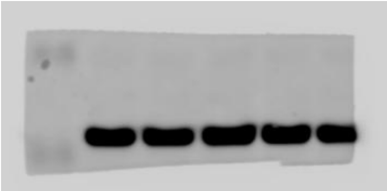	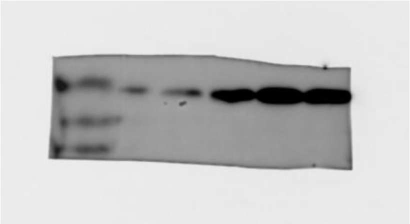
FIG3-D, anti-GAPDH	FIG3-D, anti-NLRX1
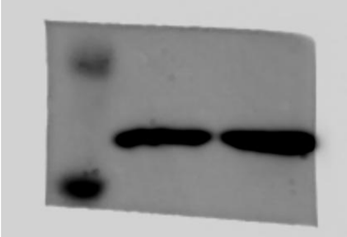	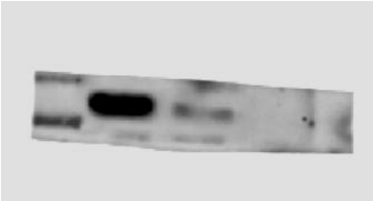
FIG4-C, anti-GAPDH	FIG4-C, anti-NLRX1
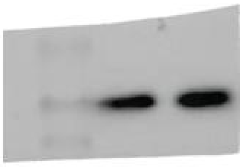	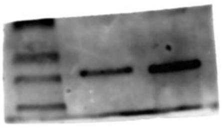
FIG4-D, anti-GAPDH	FIG4-D, anti-NLRX1
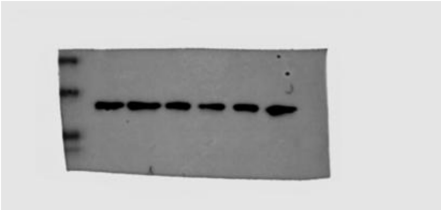	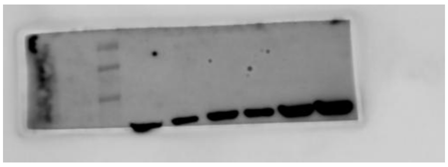
FIG6-D, anti-GAPDH	FIG6-D, anti-σc
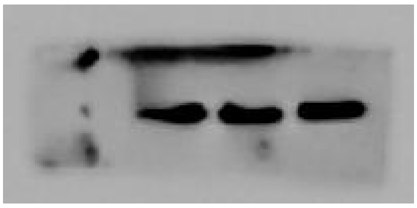	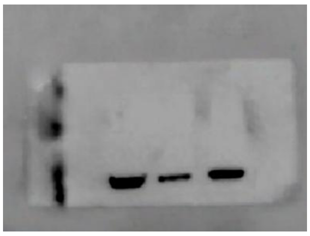
FIG8-B, anti-GAPDH	FIG-8-B, anti-NLRX1
