# Supplementary figures and images for: Essential role of the D domain of linc000889 in inhibiting avian reovirus replication
Source: Poult Sci. 2026 Jun 8;105(10):107235. doi: 10.1016/j.psj.2026.107235 (PMC13316747; doi:10.1016/j.psj.2026.107235)

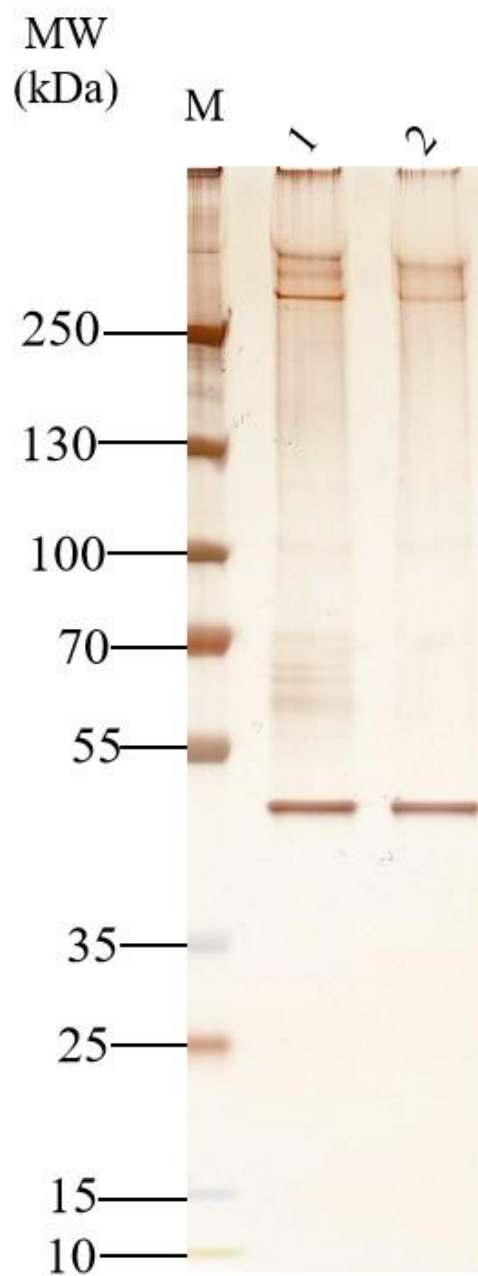

M:marker; 1: sense; 2:antisense

Supplement: Supplementary file 4 [file mmc4.pdf]
